# Hop bitter acids containing a β-carbonyl moiety prevent inflammation-induced cognitive decline via the vagus nerve and noradrenergic system

**DOI:** 10.1038/s41598-020-77034-w

**Published:** 2020-11-18

**Authors:** Yasuhisa Ano, Rena Ohya, Takahiro Yamazaki, Chika Takahashi, Yoshimasa Taniguchi, Keiji Kondo, Akihiko Takashima, Kazuyuki Uchida, Hiroyuki Nakayama

**Affiliations:** 1 Laboratory of Veterinary Pathology, Graduate School of Agricultural and Life Sciences, the University of Tokyo, Tokyo, 113-8657 Japan; 2 Kirin Central Research Institute, Kirin Holdings Company Ltd, 1-13-5 Fukuura Kanazawa-ku, Yokohama-shi, Kanagawa, 236-0004 Japan; 3 Faculty of Science, Gakushuin University, Tokyo, 171-8588 Japan

**Keywords:** Diseases of the nervous system, Neural ageing, Neuroimmunology, Peripheral nervous system

## Abstract

The prevention of age-related cognitive decline and dementia is becoming a high priority because of the rapid growth of aging populations. We have previously shown that hop bitter acids such as iso-α-acids (IAAs) and matured hop bitter acids (MHBAs) activate the vagus nerve and improve memory impairment. Moreover, supplements with MHBAs were shown to improve memory retrieval in older adults. However, the underlying mechanisms have not been entirely elucidated. We aimed to investigate the effects of MHBAs and the common β-tricarbonyl moiety on memory impairment induced by the activation of microglia and the loss of the noradrenergic system. MHBAs and a model compound with β-tricarbonyl moiety were administered to LPS-inoculated mice and 5 × FAD Alzheimer’s disease (AD) model mice, following the evaluation in behavioral tests and microglial activation. To evaluate the association of noradrenaline with MHBAs effects, mice treated with N-(2-chloroethyl)-N-ethyl-2-bromobenzylamine (DSP-4), a noradrenergic neurotoxin that selectively damages noradrenergic projections from the locus coeruleus, were subjected to the behavioral evaluation. MHBAs reduced brain inflammation and improved LPS-induced memory impairment. A model compound possessing the β-tricarbonyl moiety improved the LPS-induced memory impairment and neuronal loss via the vagus nerve. Additionally, the protective effects of MHBAs on memory impairment were attenuated by noradrenaline depletion using DSP-4. MHBAs suppressed the activation of microglia and improved the memory impairment in 5 × FAD mice, which was also attenuated by noradrenaline depletion. Treatment with MHBAs increased cholecystokinin production from the intestinal cells. Generally, cholecystokinin activates the vagal nerve, which stimulate the noradrenergic neuron in the locus ceruleus. Taken together, our results reveal that food ingredients such as hop bitter acids with a β-tricarbonyl moiety suppress microglial activation and improve memory impairment induced by inflammation or AD pathology via the activation of the gut-brain axis and noradrenergic system. Supplements with hop bitter acids, including MHBAs, might be a novel approach for the prevention of cognitive decline and dementia.

## Introduction

The number of people with cognitive decline and dementia is rapidly because of the aging society. Since there is no effective therapies for dementia, preventive approaches, including regular exercise and a healthy diet, have been receiving increasing attention. In patients with Alzheimer’s disease (AD), amyloid-β (Aβ) and phosphorylated Tau become aggregated and are deposited as senile plaques and neurofibrillary tangles (NFTs), respectively^1,2^. It is showed that the accumulation of Aβ and phosphorylated Tau in the brain induces brain inflammation and exacerbates neurological pathologies and memory impairment^3–5^. Brain inflammation is regulated by microglia, which have an immunological function producing cytokines and chemokines and removing pathogens, waste products and old synapses via phagocytosis^6^. In patients with AD, the infiltration of activated microglia around senile plaques and NFTs is a prominent feature^4^, and activated microglia are associated with neurotoxic effects and disease exacerbation^7^. Therefore, regulating the activation of microglia has been gaining increasing attention for the preventive and therapeutic targets of dementia^7,8^.

We have previously demonstrated that iso-α-acids (IAAs), hop-derived bitter acids in beer, prevent brain inflammation and memory impairment in Alzheimer’s disease model mice and aged mice^9–13^. IAAs are produced from α-acids in hops during the brewing process for beer. IAAs are potent agonists for the bitter taste receptors TAS2Rs (T2Rs) which are abundant in enteroendocrine cells^14–17^. The activation of T2Rs increases Ca^2+^ and leads to the release of cholecystokinin (CCK). CCK transmits signals to the brain via its receptors on sensory fibers of the vagus nerve and modifies food intake^18–21^.

We previously found that matured hop-derived bitter acids (MHBAs) activate the vagus nerve and noradrenergic system in the brain, thus improving memory impairment^22,23^. MHBAs are also derived from α- and β-acids and show mild bitterness^24,25^. In addition, we demonstrated that supplements with MHBAs improve cognitive function, including memory retrieval and attention, in older adults^26^. These findings indicate that activation of the vagus nerve by food ingredients is beneficial for preventing cognitive decline and dementia. Interestingly IAAs and MHBAs have common chemical structures, which include a β-tricarbonyl moiety, possibly contributing to vagal activation^24,27,28^.

Recent investigations have shown that vagus nerve stimulation (VNS) is a therapeutic approach for brain disorders. VNS has been used to treat patients with epilepsy and those with treatment-resistant depression or severe, recurrent unipolar and bipolar depression^29^. However, since surgical operation is required for treatment with VNS, VNS is not the first approach for treatment. VNS by food ingredients is safer and easier to practice in daily life. Both IAAs and MHBAs improve memory impairment via the vagus nerve^30^, although common underlying mechanisms have not been entirely elucidated yet. In the present study, we investigated the effects of MHBAs and the common β-tricarbonyl moiety on memory impairment induced by the activation of microglia and the loss of the noradrenergic system.

## Methods

### Materials

**Figure 1:**
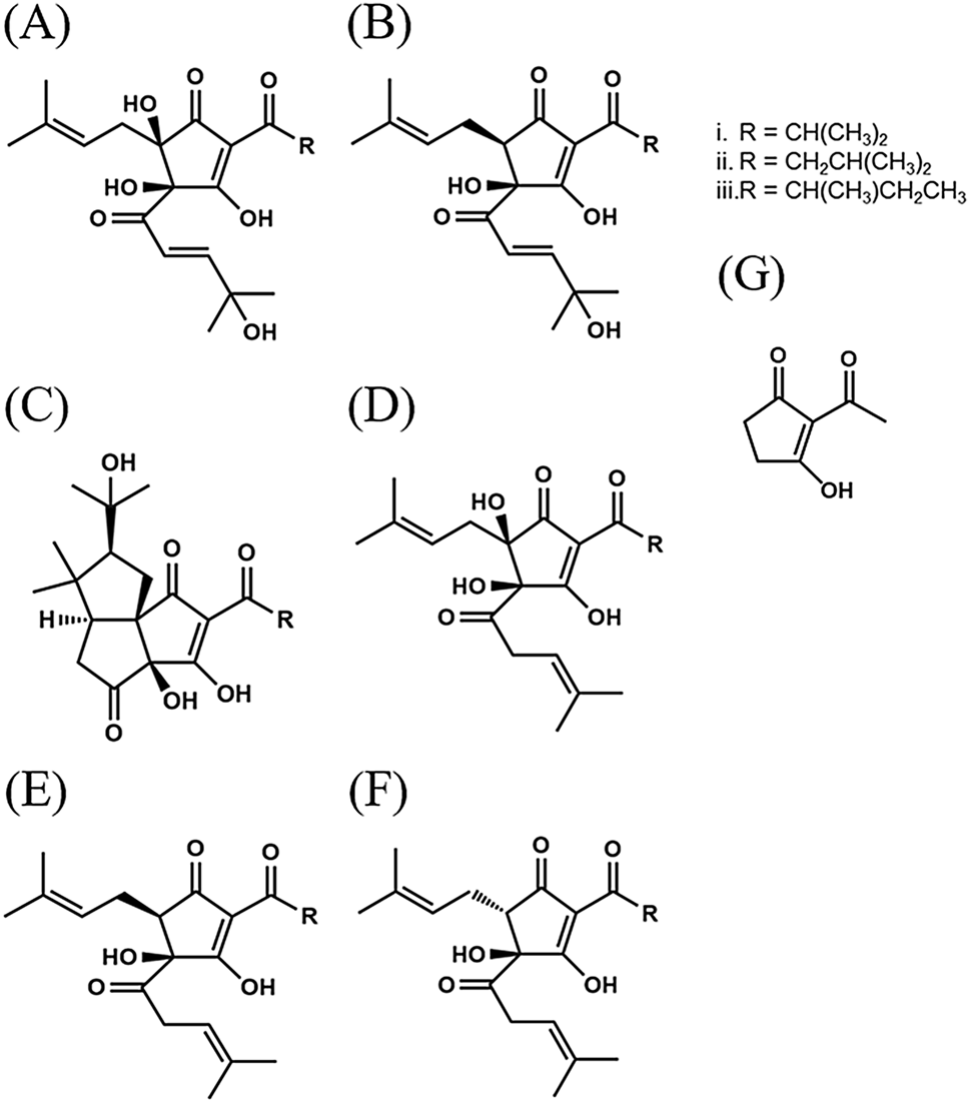
Chemical structures of hop bitter acids. (**A**) 4′-hydroxyallohumulinones. (**B**) 4′-hydroxyallo-cis-isohumulones. (**C**) tricyclooxyisohumulones A. (**D**, humulinones. (**E**), cis-iso-α-acids. (**F**) trans-iso-α-acids. (**G**) 2-acetyl-3-hydroxy-2-cyclopenten-1-one, a model compound containing a β-tricarbonyl moiety.

MHBAs and their major components [4′-hydroxyallohumulinones (HAH); 4′-hydroxyalloisohumulones (HAIH); tricyclooxyisohumulones A (TCOIH-A), and humulinones] were prepared from hop pellets, as previously described^24,25,27,31^ (Fig. 1A-D). MHBAs and IAAs (Fig. 1E and F) contain a β-tricarbonyl moiety^24^. A compound possessing the β-tricarbonyl moiety (2-acetyl-3-hydroxy-2-cyclopenten-1-one, AHC; Fig. 1G) was prepared according to a previously reported method^32^ and was used as a model compound in the present study.

### Animals

Six-week-old Crl:CD1(ICR) male mice were purchased from Charles River Japan (Tokyo, Japan) and maintained at Kirin Holdings Co. Ltd. Vagotomized and sham-operated male ICR mice were prepared in the laboratory of Charles River Japan Inc.; vagotomy was performed at 5 weeks of age, and the experiments were performed when the mice were 6 weeks old. All experiments were approved by the Animal Experiment Committee of Kirin Holdings Co. Ltd. and conducted in strict accordance with their guidelines. The ethical approval numbers were AN10339-Z00 and AN10569-Z00.

The AD B6SJL-Tg model mice (APPSwFlLon, PSEN1*M146L*L286V, https://jaxmice.jax.org/strain/006554.html,^33^), hereafter referred to as 5 × FAD transgenic mice, were purchased from Jackson Laboratory (Sacramento, CA, USA) and were maintained by crossing hemizygous transgenic mice with B6SJLF1/J mice in the experimental facility of the University of Tokyo. 5 × FAD transgenic mice with five AD-linked mutations, which overexpressed human amyloid precursor protein (APP) with the mutations of K670N, M671L, I716V, and V717I Familial Alzheimer's Disease (FAD) and human presenilin 1 (PS1) with the mutations of M146L and L286V. Non-transgenic littermates were used as wild-type controls. All experiments conducted in the present study were approved by the Animal Care and Use Committee of the Graduate School of Agricultural and Life Sciences, University of Tokyo, and performed in accordance with the guidelines (Approval No, P15-042). There were no significant differences in body weight and food consumption among different mouse groups at 2.5 months of age. After behavioral evaluations, mice were euthanized, and the brains were removed, as described in the following sections.

All efforts were made to minimize animal suffering. The present study was conducted from April 2016 to July 2018. Mice were maintained at 23 °C ± 1 °C under constant 12 h light and dark cycles. Mice aged < 3 months were fed AIN-93G (Oriental Yeast, Tokyo, Japan), and those aged ≥ 3 months were fed AIN-93 M (Oriental Yeast).

### Lipopolysaccharide (LPS)-induced neural inflammation

ICR mice were orally administered with 0 or 1 mg/kg of MHBAs, HAIH, HAH, and TCOIH-A dissolved in distilled water (10 mL/kg) once a day for 3 days. Crl:CD1 mice were orally administered with 0, 0.3, 1, or 3 mg/kg of AHC dissolved in distilled water (10 mL/kg) once a day for 3 days. One hour after the last administration, mice were deeply anesthetized with sodium pentobarbital (Somnopentyl; Kyoritsu Seiyaku, Tokyo, Japan) and intraventricularly injected with 10 μg of LPS (L7895, Sigma-Aldrich, St. Louis, MO, USA), in accordance with previous work^34,35^. Briefly, LPS dissolved in PBS or PBS (for sham-operated controls) was injected into the cerebral ventricle in both hemispheres, as previously described^35^. A micro-syringe with a 27-gauge stainless steel needle, 2 mm in length, was used for the microinjection. The needle was inserted unilaterally, 1 mm to the right and left of the midline point equidistant from each eye, in both the left and right hemispheres, at an equal distance between the eyes and ears, and perpendicular to the plane of the skull. LPS was delivered gradually within 30 s. The needle was withdrawn after waiting for 30 s.

To evaluate spatial working memory, the mice were orally administered with the compounds 24 h after LPS treatment and subjected to the spontaneous alternation test 1 h after the last oral administration, as described below.

To evaluate object recognition memory, the mice were orally administered with the compounds 24 h after LPS treatment and subjected to the novel object recognition test (NORT) 1 h after the oral administrations in the acquisition and retention steps, as described below.

To evaluate the inflammatory response, the hippocampus and cortex of the corresponding hemisphere were removed 24 h after LPS treatment and homogenized in Tris-buffered saline (Wako, Tokyo, Japan) containing a protease inhibitor cocktail (BioVision, CA, USA) using a multi-beads shocker (Yasui Kikai, Osaka, Japan). After centrifugation at 50,000 × g for 20 min, the supernatants were collected. The total protein concentration of each supernatant was measured using a BCA protein assay kit (ThermoScientific, Yokohama, Japan). To evaluate inflammation in the brain, the amount of tumor necrosis factor (TNF)-α in the supernatants was quantified using enzyme-linked immunosorbent assay (ELISA; eBiosciences, CA, USA). The other hemisphere was used for morphological analysis of the dendrites. Brain sections at bregma − 2.06 mm were prepared and stained using the FD Rapid GolgiStain Kit (FD Neuro Technologies, MD, USA), following the manufacturer’s instructions. Spines were counted within the dentate gyrus (DG), CA1, and locus coeruleus (LC); dendrites were counted starting from their point of origin, from the primary dendrite, as previously described^34^. For spine density measurements, all areas containing 50–100 μm of secondary dendrites from all neurons, where the image was clearly captured, were used.

### Noradrenaline neuron depletion

ICR mice were treated intraperitoneally with saline or 50 mg/kg N-(2-chloroethyl)-N-ethyl-2-bromobenzylamine (DSP-4; Sigma-Aldrich, St. Louis, MO, USA) dissolved in saline. Five days after DSP-4 treatment, mice were orally administered with MHBAs at 0, 1, or 3 mg/kg once a day for 5 days, and on day 8 after DSP-4 treatment, they were intraventricularly injected with 10 μg of LPS, as described above. At 9 and 10 days after DSP-4 treatment, mice were subjected to the NORT. In this test, mice were orally administered with MHBAs 1 h before each acquisition and retention step.

### Assessment of microglia activation in AD transgenic mice

To deplete noradrenaline neurons in AD transgenic mice, 2.5-month-old transgenic 5 × FAD and wild-type male mice were treated intraperitoneally with saline or 50 mg/kg DSP-4 dissolved in saline at 2.5, 2.7, 3.5, 4.5, and 5.5 months of age. Mice were also fed with a diet that contained or did not contain 0.05% w/w MHBAs for 3.5 months (N = 10 per group, 8 groups). Six-month-old transgenic 5 × FAD and wild-type male mice were subjected to the NORT.

Following the behavioral evaluation, to evaluate microglia activation, the right-brain hemisphere (N = 5 per group) was removed, and complement receptor (CD)11b-positive microglia were isolated and used for flow cytometry analysis, as described in previous studies^12,20,21^. Brain cells were obtained by papain treatment using the Neural Tissue Dissociation Kit (P) (Miltenyi Biotec, MA, USA). Cells were treated with 2 μg/mL of anti-CD11b antibody conjugated with microbeads (Miltenyi Biotec), and CD11b-positive cells were isolated by magnetic cell sorting.

To analyze intracellular cytokine production using a FACS Canto II flow cytometer (BD Biosciences), isolated primary microglia were cultured in DMEM/F-12 medium (Gibco, CA, USA) with 10% fetal calf serum (Gibco) and 100 U/mL penicillium/streptomycin (Sigma-Aldrich, MO, USA) and treated with a leukocyte activation cocktail using BD GolgiPlug (BD Biosciences) for 12 h. The cells were analyzed using a flow cytometer after the fixation with a BD Cytofix/Cytoperm Fixation/Permeabilization kit (BD Biosciences) and subsequent staining with the antibodies: anti- TNF-α-APC (MP6-XT22, BD Pharmingen, CA, USA), and anti-CD11b-APC-Cy7 (M1/70, BD Pharmingen).

To measure the phagocytotic activity of 6-carboxyfluorescein-labeled Aβ1–42 (Aβ-FAM; AnaSpec, Fremont, CA), as previously described^36^, microglial cells isolated from newborn mice were plated at a density of 50,000 cells/well in poly-D-lysine-coated 96-well plates and incubated with 500 nM Aβ-FAM for 24 h. After the medium was removed, extracellular Aβ-FAM was quenched with 0.2% trypan blue, pH 4.4. Cellular fluorescence intensity of the areas of 5 wells/sample was measured at 485-nm excitation/535-nm emission using a plate reader (Molecular Devices, Sunnyvale, CA).

To measure the level of noradrenaline in the brainstem, according to a previous report^22^, the tissue was homogenized in 0.2 M perchloric acid (Wako) containing 100 μM EDTA•2Na (Sigma-Aldrich). After centrifugation, the supernatant was analyzed by high performance liquid chromatography (HPLC) using an EICOMPAK SC-5ODS column and a PREPAK column (Eicom, Kyoto, Japan) with an electrochemical detection (ECD) unit. The mobile phase consisted of 83% 0.1 M acetic acid in a citric acid buffer (pH 3.5), 17% methanol (Wako), 190 mg/mL sodium 1-octane sulfonate sodium (Wako), and 5 mg/mL EDTA•2Na. For ECD, the applied voltage was 750 mV vs that of an Ag/AgCl reference electrode.

### Spontaneous alteration test

The spontaneous alternation test was conducted in accordance with our previous report^7^. The apparatus comprised a Y-maze, i.e., a three-arm maze with equal angles among all arms (25-cm long × 5-cm wide × 20-cm high). The maze walls were constructed from dark black, polyvinyl plastic. Each mouse was initially placed in one arm, and the sequence and number of arm entries were counted for 8 min. The alternation score (%) for each mouse was defined as the ratio of the actual number of alternations to the possible number (defined as the total number of arm entries minus two) multiplied by 100 as follows: % Alternation = [(Number of alternations) / (Total arm entries − 2)] × 100.

### Novel object recognition test (NORT)

The NORT was performed according the previous reports^9,37^. Briefly, in the acquisition step, a pair of triangle poles (4.5 × 4.5 × 4.5 cm^3^) or pyramids (4.5 × 4.5 × 4.5 cm^3^) was used and in the retention step, a pair of poles or pyramids and a golf ball (4.5-cm diameter) were used. Each object was counterbalanced for the preference (data not shown). In all trials, the objects were placed 7.5 cm × 7.5 cm apart from the corner of a polyvinyl chloride box (40 × 40 × 40 cm^3^) without a roof. In the acquisition trial, each mouse freely explored the box for 10 min and 24 h after the acquisition trial, the mouse was allowed to explore the box with the novel and familiar objects for 5 min. The discrimination index was calculated by (novel object exploration time – familiar object exploration time) / (total exploration time).

### Open field test

To evaluate activity in a novel place, mice were placed in an open box (40 × 40 × 40 cm^3^, gray polyvinyl chloride), without a roof, for 5 min. Mouse activity was monitored using the SMART video tracking software (PanLab Harvard Apparatus, MA, USA).

### Cell culture

The STC-1 cell line is derived from an intestinal endocrine tumor obtained from double-transgenic mice expressing the rat insulin promoter linked to the simian virus 40 large T antigen and polyoma small T antigen ^18^. The cell line was purchased from ATCC (Manassas, VA, USA) and was maintained in DMEM containing 10% fetal bovine serum, 100 U/mL penicillin, and 100 μg/mL streptomycin (Gibco, Grand Island, NY, USA) at 37 °C in 5% CO_2_/air humidity.

CCK release from STC-1 cells was determined based on previous reports^38,39^. Cells were grown overnight in 24-well plates (5 × 10^5^ cells/well), washed and exposed to 0, 30, 100, or 300 μM MHBAs or the β-tricarbonyl model compound dissolved in a culture medium containing 0.3% DMSO for 60 min at 37 °C in 5% CO2/air humidity. To determine the time dependency of CCK release, the incubation was performed for 90 min, and CCK was detected at 60 min of incubation. Measurements of CCK levels were performed using a commercial ELISA kit (Phoenix Pharmaceuticals Inc., Belmont, CA, USA), according to manufacturer's instructions.

### Statistical analysis

Data acquired in the experiments were analyzed using the Student’s t-tests, Dunnett’s test, one-way analysis of variance (ANOVA), followed by Tukey’s multiple comparisons test or analyzed using a two-way analysis of variance followed by Tukey’s multiple comparisons test which were described in figure legends. *P* < 0.05 was considered statistically significant. All statistical analyses were performed using GraphPad Prism 7 (GraphPad Software, Inc., CA, USA).

### Ethics approval

All experiments with Crl:CD1 mice were approved by the Animal Experiment Committee of Kirin Holdings Co. Ltd. and conducted in strict accordance with their guidelines. The ethical approval numbers were AN10339-Z00 and AN10569-Z00. All experiments with 5 × FAD mice were approved by the Animal Care and Use Committee of the Graduate School of Agricultural and Life Sciences, University of Tokyo, and conducted in strict accordance with their guidelines (Approval No, P15-042).

### Consent for publication

Not applicable.

## Results

### Effects of MHBAs on memory impairment induced by LPS

**Figure 2:**
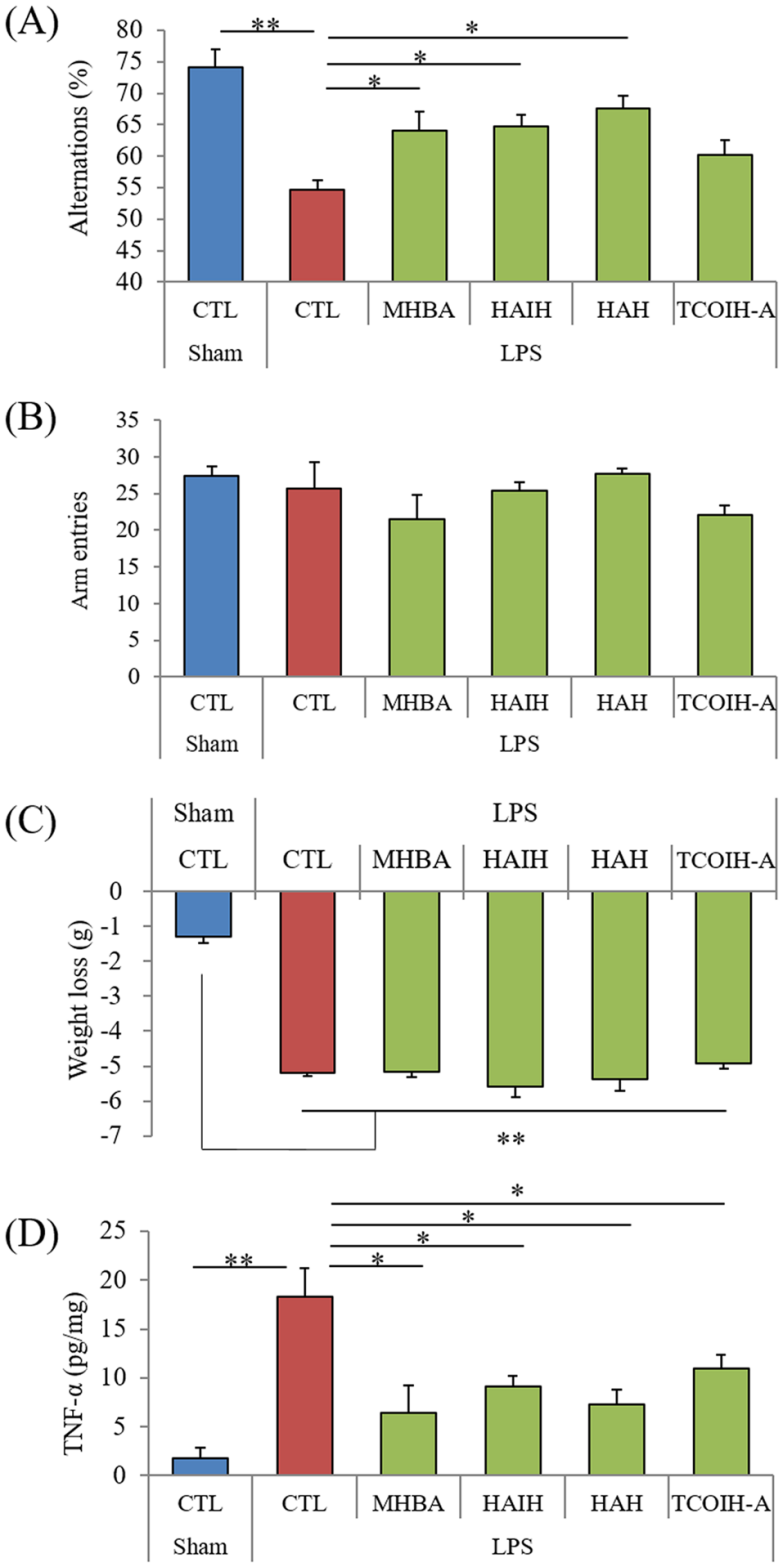
Effects of MHBAs on memory impairment and cytokine production in LPS-treated mice. Crl:CD1 mice were orally administered 0 or 1 mg/kg of MHBAs, HAIH, HAH, and TCOIH-A for 3 days, and intracerebroventricularly injected with 10 μg of LPS or saline. Mice were subjected to the spontaneous alternation test, and the levels of TNF-α in the hippocampus were measured. **A** and **B**, Alternations (**A**) and arm entries (**B**) in the spontaneous alternation test. (**C**) Body weight changes by LPS treatment. (**D**) The levels of TNF-α in the hippocampus. Data are presented as mean ± standard error of the mean (7 mice per group). *P*-values shown in the graph were calculated by one-way analysis of variance followed by the Tukey–Kramer test. *p < 0.05, **p < 0.01. MHBAs, matured hop bitter acids; LPS, lipopolysaccharide; HAIH, 4′-hydroxyallo-cis-isohumulones; HAH, 4′-hydroxyallohumulinones; TCOIH-A, tricyclooxyisohumulones A; TNF-α, tumor necrosis factor α; CTL, control.

The effects of MHBAs on memory impairment induced by inflammation were evaluated using an LPS-inoculated mouse model (N = 7 per group). Alternations in the spontaneous alternation test were significantly fewer in mice inoculated with LPS than in sham-injected mice (Fig. 2A). The number of arm entries was not different between groups (Fig. 2B), whereas the body weight was significantly reduced after treatment with LPS (Fig. 2C). The alternations in LPS-inoculated mice administered with MHBA, HAIH, and HAH were significantly more than those in control LPS-inoculated mice (*p* = 0.046, 0.039, 0.035, respectively, Fig. 2A). The number of arm entries (Fig. 2B) and weight loss (Fig. 2C) were not affected by these treatments. The levels of TNF-α in the hippocampus were significantly higher in mice inoculated with LPS than in sham-treated mice and were significantly reduced after administration with MHBA, HAIH, HAH, and TCOIH-A (*p* = 0.034, 0.021, 0.025, 0.031, respectively, Fig. 2D). (P < 0.05, one-way ANOVA [F(dFn, dFd) = F(5, 36) = 7.57]; Tukey–Kramer comparison was performed as post-hoc test).

These results indicate that MHBAs and their components reduce TNF-αproduction and improve the LPS-induced reduction in alternations.

### Effects of the β-tricarbonyl moiety of MHBAs on memory impairment in mice

The effects of AHC, a β-tricarbonyl moiety, on inflammatory-related memory impairment were evaluated (N = 7–10 per group).

**Figure 3:**
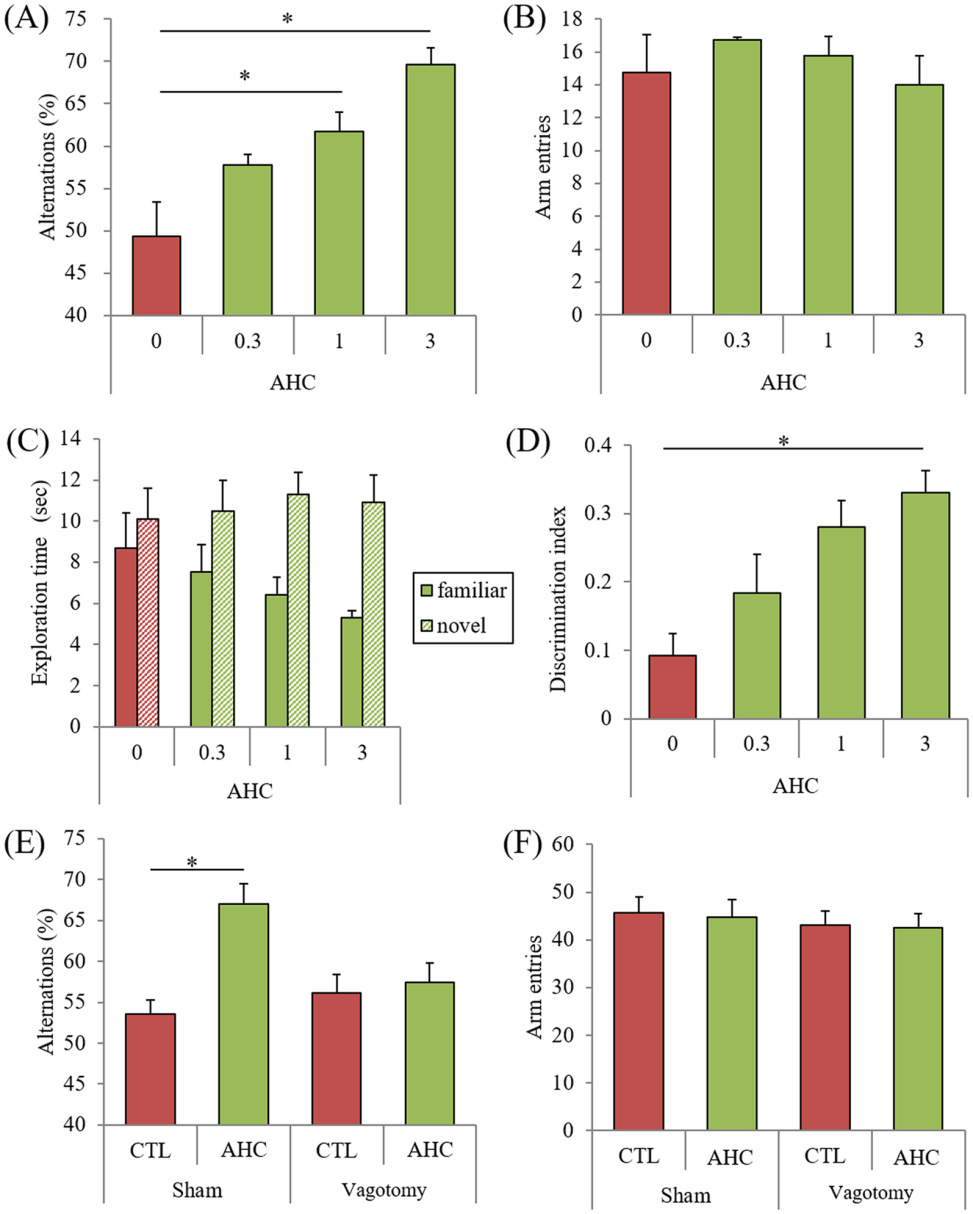
Effects of the β-tricarbonyl moiety compound on memory impairment. (**A**)–(**D**), Crl:CD1 mice were orally administered with 0, 0.3, 1, or 3 mg/kg of AHC for 3 days, and intracerebroventricularly injected with 10 μg of LPS. Mice were subjected to the spontaneous alternation test one day after LPS treatment and to the novel object recognition test 2 days after LPS treatment. (**A)** and (**B)**, Alternations (**A**) and arm entries (**B**) in the spontaneous alternation test. C and D, Time of exploring novel or familiar objects (**C**) and discrimination index in the novel object recognition test (**D**). (**E**) and (**F**) Vagotomized or sham-operated Crl:CD1 mice were administered 0 or 1 mg/kg of AHC for 3 days, and intracerebroventricularly injected with 10 μg of LPS. Mice were subjected to the spontaneous alternation test. Data are presented as mean ± standard error of the mean (7–10 mice per group). *P*-values shown in the graph were calculated by Dunnett’s test (A-D); two-way analysis of variance followed by the Tukey–Kramer test (E and F). *p < 0.05. AHC, 2-acetyl-3-hydroxy-2-cyclopenten-1-one; LPS, lipopolysaccharide; CTL, control.

In the spontaneous alternation test, used to evaluate spatial working memory, alternations were significantly higher in mice administered with 1 and 3 mg/kg AHC than in control mice (*P* = 0.045, 0.040, respectively, Fig. 3A), while the number of arm entries was not changed (Fig. 3B).

In the NORT, used to evaluate episodic object recognition memory, the percent time exploring the novel object was significantly higher in mice administered with 3 mg/kg AHC than in control mice (Fig. 3C). The discrimination index was also significantly higher in mice administered with 3 mg/kg AHC (*p* = 0.041, Fig. 3D).

The significant increase in spontaneous alternations by the administration of 1 mg/kg AHC was attenuated in vagotomized mice but not in sham-operated mice (*P* < 0.05, Vagotomy factor and AHC factor main effects [F(dFn, dFd) = F(1, 36) = 4.64 and F(1, 36) = 5.81] and Vagotomy × AHC interaction [F(1, 36) = 1.31], two-way ANOVA; *P* = 0.040, designated comparison, Tukey–Kramer post-hoc test) (Fig. 3E).The number of arm entries was not changed among groups (Fig. 3F).

### Effects of the β-tricarbonyl moiety of MHBAs on neuronal dendrites

To evaluate the effects of the β-tricarbonyl moiety on neuronal dendrites, the number of spines was measured in mice inoculated with LPS (N = 7 per group).

**Figure 4:**
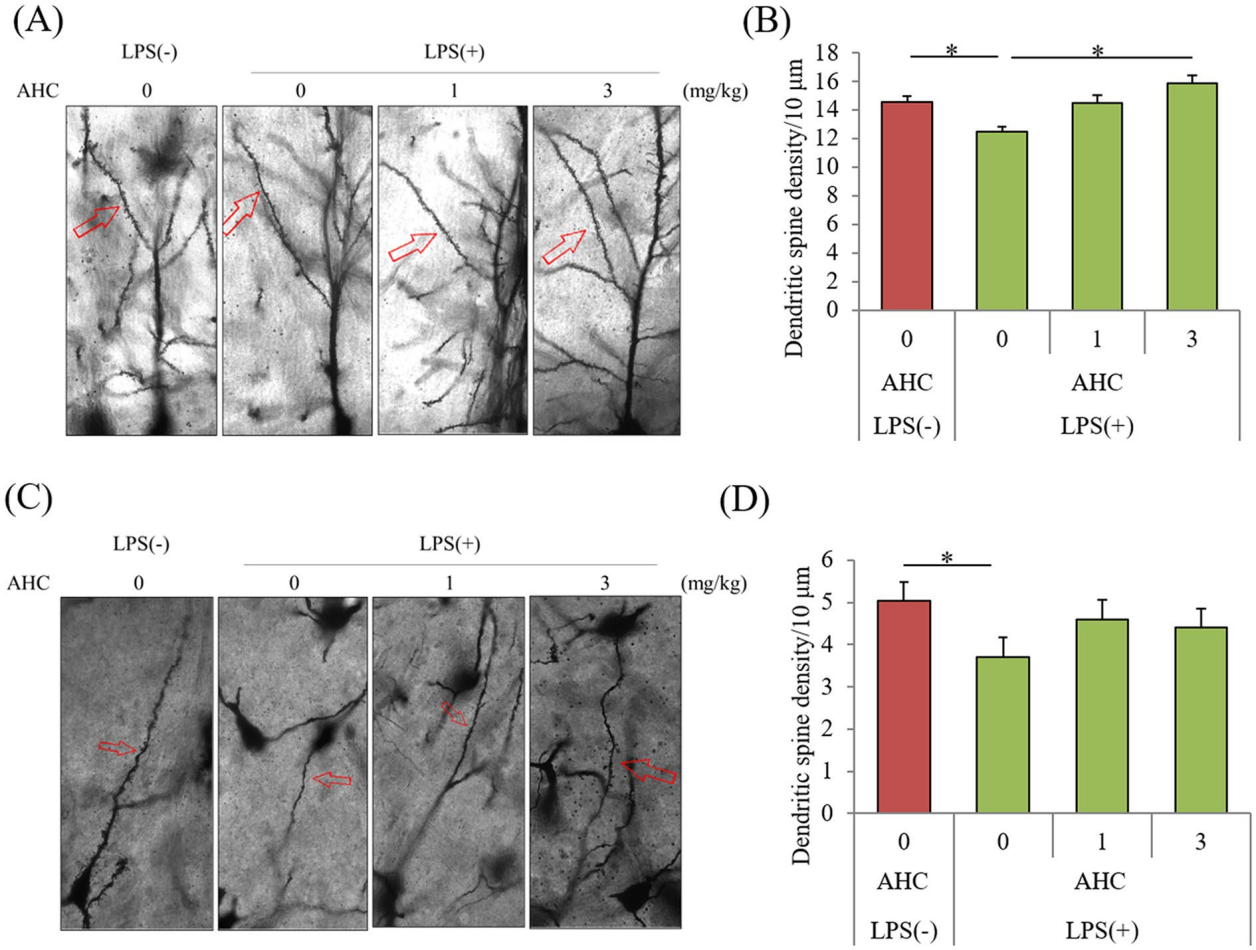
Effects of the β-tricarbonyl moiety compound on LPS-induced changes in dendritic spine density. Crl:CD1 mice were orally administered with 0, 1, or 3 mg/kg of AHC for 3 days, and intracerebroventricularly injected with 10 μg of LPS. At 3 days after LPS treatment, the brains were subjected to Golgi staining. (**A**) and (**C**) Representative images of Golgi staining in CA1 of hippocampus and locus coeruleus (LC), respectively. (**B**) and (**D**) Number of dendritic spines per 10 μm in CA1 (**B**) and LC (**D**). Data are presented as mean ± standard error (7 mice per group). The *p* values shown were calculated by one-way analysis of variance followed by the Tukey–Kramer test. *p < 0.05. AHC, 2-acetyl-3-hydroxy-2-cyclopenten-1-one; LPS, lipopolysaccharide.

The number of dendritic spines in the CA1 region of the hippocampus was significantly reduced in the mice treated with LPS compared with that in sham-treated mice but was significantly increased upon treatment with 3 mg/kg AHC (*p* = 0.039, Fig. 4A and C). (P < 0.05, one-way ANOVA [F(dFn, dFd) = F(3,28) = 7.29]; Tukey–Kramer comparison was performed as post-hoc test) The number of dendritic spines in the DG was not changed by the treatment with LPS (data not shown), whereas that in the LC was significantly reduced compared to the number in sham-treated mice; this reduction by LPS was not observed in mice administered with AHC (Fig. 4B and D).

### MHBA effects on inflammation-induced memory impairment are mediated by the noradrenergic system

To examine whether noradrenaline mediates the effects of MHBAs on memory improvement, mice orally administered with MHBAs were treated with DSP-4 and subsequently inoculated with LPS (N = 8–10 per group). In the NORT, the discrimination index was significantly higher in mice administered with 1 mg/kg and 3 mg/kg MHBAs than in control mice (p = 0.039 and 0.041, respectively); this significant improvement was attenuated by treatment with DSP-4, a selective neurotoxin for noradrenaline neurons (*P* < 0.05, DSP-4 factor and MHBA factor main effects [F(dFn, dFd) = F(1, 51) = 1.00 and F(2, 51) = 7.04] and DSP-4 × MHBA interaction [F(2,51) = 1.62], two-way ANOVA; designated comparison, Tukey–Kramer post-hoc test) (Supplementary Figure S1B). The total distance traveled in the open field remained unchanged among the groups (Supplementary Figure S1C).

### Noradrenaline mediates MHBA effects on memory impairment in 5 × FAD mice

**Figure 5:**
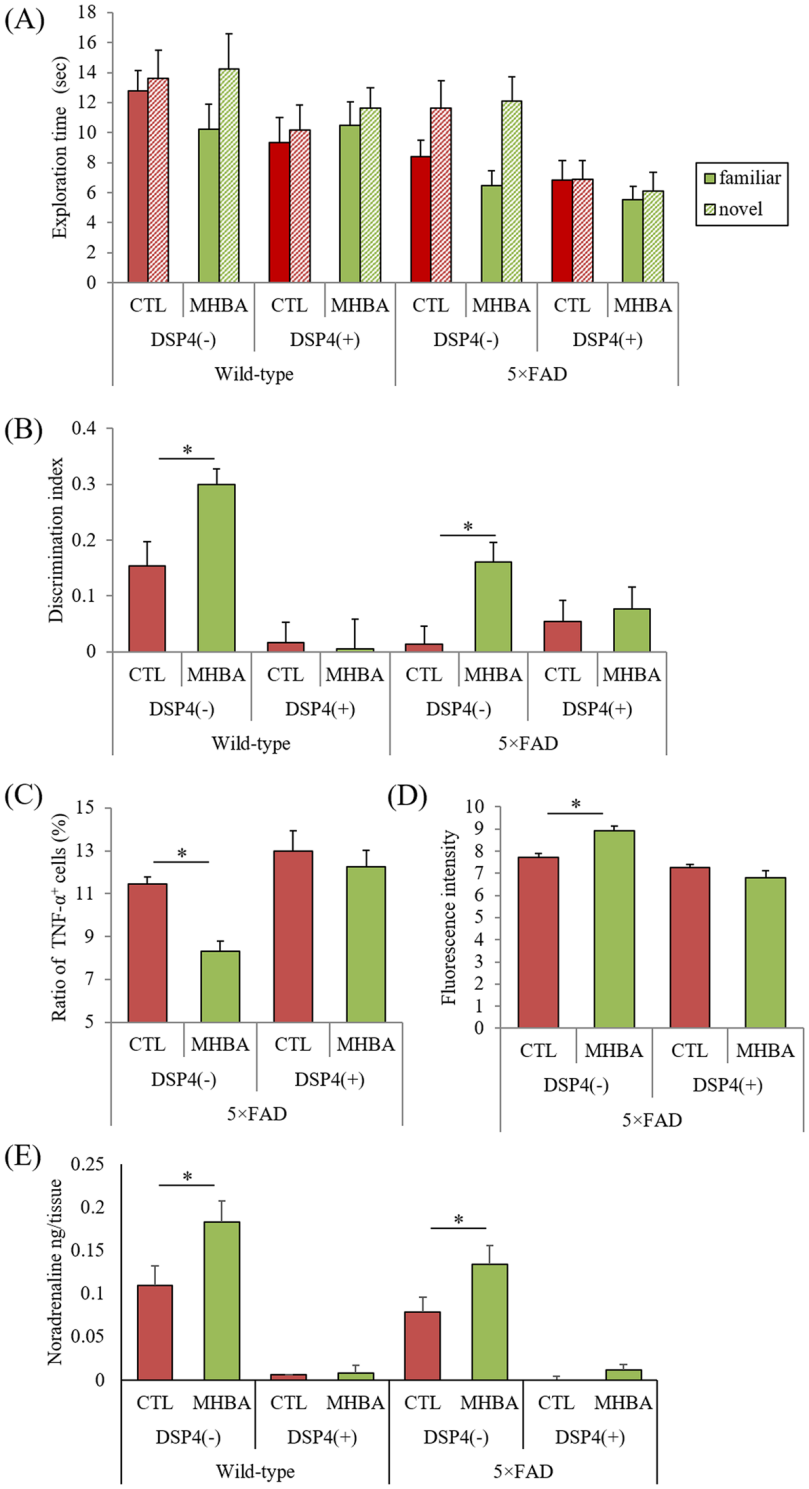
Effects of MHBAs on microglial activation in 5 × FAD mice. Transgenic 5 × FAD and wild-type male mice aged 2.5 months were fed a diet with or without 0.05% w/w MHBAs for 3.5 months. Mice were treated intraperitoneally with saline or 50 mg/kg DSP-4 at 2.5, 2.7, 3.5, 4.5, and 5.5-months of age. (A) Time of exploring novel or familiar objects. (**B**) Discrimination index in the novel object recognition test. (**C**) and (**D**) Isolated microglia were treated with a leukocyte activation cocktail and analyzed using flow cytometry to measure intracellular cytokines. Isolated microglia were incubated with 500 nM FAM-labeled Aβ, and the fluorescence intensity of the entire area of 5 wells/sample was measured at 485-nm excitation/535-nm emission. The graph in C shows the percentage of TNF-α-producing cells among CD11b-positive cells. The graph in D shows the fluorescent intensity of Aβ-FAM-treated microglia. E, Noradrenaline levels in the brainstem measured using a high-performance liquid chromatography-electrochemical detection system. Data are mean ± standard error (10 mice per group). The *p* values shown were calculated by Two-way ANOVA and Tukey–Kramer as a post-hoc test *p < 0.05. MHBAs, matured hop bitter acids; DSP-4, N-(2-chloroethyl)-N-ethyl-2-bromobenzylamine; TNF-α, tumor necrosis factor-α; Aβ, amyloid-beta; CTL, control.

To assess whether noradrenaline also mediates the effects of MHBAs on memory improvement in AD model mice, 5 × FAD mice treated with DSP-4 were subjected to the NORT (N = 8–10 per group). In wild-type mice, the discrimination index significantly increased after MHBA treatment, and this improvement was attenuated by treatment with DSP-4 (p = 0.034, Fig. 5A and B). In 5 × FAD mice, MHBA treatment also significantly increased the discrimination index compared with that after control treatment (*p* = 0.043), and this improvement was also attenuated by DSP-4 treatment (*P* < 0.05, DSP-4 factor and MHBA factor main effects [F(dFn, dFd) = F(1, 33) = 0.13 and F(1, 33) = 4.36] and DSP-4 × MHBA interaction [F(1, 33) = 2.41], two-way ANOVA;, designated comparison, Tukey–Kramer post-hoc test) (Fig. 5B).

Next, the effects of MHBAs on microglia activation were measured. Flow cytometry analysis showed that the percentage of TNF-α-producing cells to CD11b-positive cells was significantly lower in 5 × FAD mice administered with MHBAs than in control 5 × FAD mice, a reduction that was attenuated in 5 × FAD mice treated with DSP-4 (*P* < 0.05, DSP-4 factor and MHBA factor main effects [F(dFn, dFd) = F(1, 16) = 28.17 and F(1, 16) = 58.72] and DSP-4 × MHBA interaction [F(1, 16) = 11.58], two-way ANOVA; *P* = 0.031, designated comparison, Tukey–Kramer post-hoc test) (Fig. 5C).

The Aβ phagocytosis of CD11b-positive cells was measured ex vivo using Aβ labeled with FAM. The fluorescent intensity was significantly increased in 5 × FAD mice administered with MHBAs compared with that in control 5 × FAD mice, indicating that the phagocytosis of Aβ in CD11b-positive cells was increased by MHBA administration; however, this enhancement of Aβ phagocytosis in CD11b-positive cells was attenuated by the treatment with DSP-4 (*P* < 0.05, DSP-4 factor and MHBA factor main effects [F(dFn, dFd) = F(1, 16) = 6.69 and F(1, 16) = 4.64] and DSP-4 × MHBA interaction [F(1, 16) = 13.01], two-way ANOVA; *P* = 0.040, designated comparison, Tukey–Kramer post-hoc test) (Fig. 5D).

The levels of noradrenaline were measured using an HPLC-ECD system. Noradrenaline levels were significantly higher in wild-type mice and 5 × FAD mice administered with MHBAs than in the respective control mice (p = 0.041 and 0.047, respectively), while DSP-4 treatment significantly reduced the levels of noradrenaline (*P* < 0.05, DSP-4 factor and MHBA factor main effects [F(dFn, dFd) = F(1, 33) = 2.30 and F(1, 33) = 20.25] and DSP-4 × MHBA interaction [F(1, 33) = 0.93], two-way ANOVA; designated comparison, Tukey–Kramer post-hoc test) (Fig. 5E).

### Effects of MHBAs on cholecystokinin production by intestinal cells

**Figure 6:**
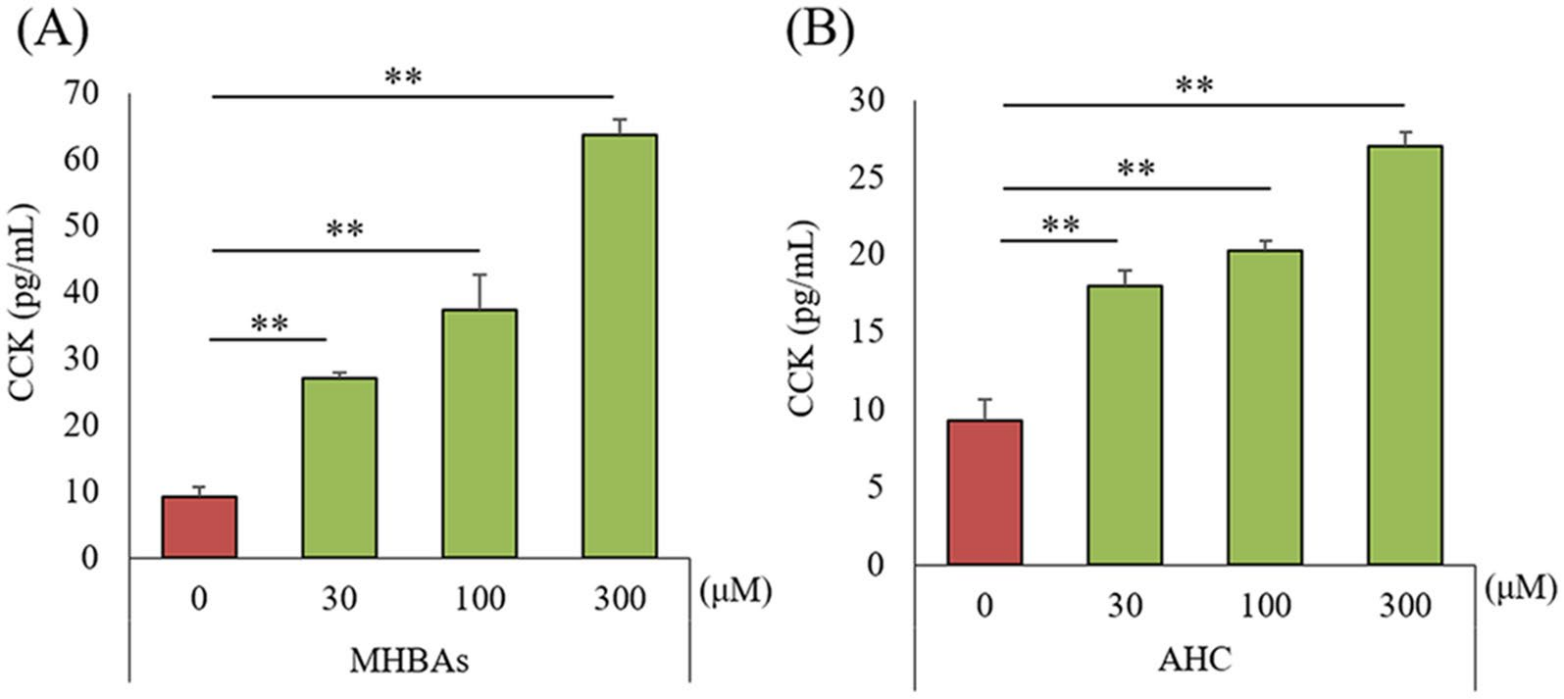
Effects of MHBAs and β-tricarbonyl moiety on CCK release from STC-1 cells. A and B, CCK levels were measured 60 min after treatment of STC-1 cells with 0, 30, 100, or 300 μM MHBAs or AHC. Data are mean ± standard error (3 wells). The *p* values shown were calculated by Dunnett’s test. **p < 0.01. MHBAs, matured hop bitter acids; CCK, cholecystokinin; AHC, 2-acetyl-3-hydroxy-2-cyclopenten-1-one.

To evaluate the effects of MHBAs on CCK production, STC-1 cells were treated with MHBAs and the β-tricarbonyl moiety compound (AHC), and the levels of CCK in the supernatant were measured. The levels of CCK were significantly increased by the treatment with 30, 100, and 300 μM MHBAs and AHC treatment (Fig. 6A and B).

## Discussion

This is the first study to show that MHBAs containing β-tricarbonyl moiety improved memory impairment caused by brain inflammation and to indicate the association between the norepinephrine system and vagal nerve activated by MHBAs and memory improvement.

Previous studies have shown that MHBAs activate the vagus nerve and increase the levels of noradrenaline, resulting in the improvement of memory impairment^22^. A clinical trial showed that supplements with MHBAs improved cognitive function in older adults with subjective cognitive decline^26^. We have previously shown that MHBAs are associated with the vagus nerve and nicotinic acetylcholine receptors to improve cognitive function^22,23^; however, the mechanisms underlying the effects of MHBAs on cognitive decline have not been entirely investigated. In the current study, we found that MHBAs prevented the inflammation-related cognitive decline and that the β-tricarbonyl moiety, which is common in the structure of MHBAs and IAAs, contributed to these effects via the activation of the vagus nerve. Moreover, MHBAs suppressed the activation of microglia and memory impairment observed in AD model mice, an effect that was mediated by the noradrenergic system.

Recent investigations have shown that VNS is a therapeutic approach for brain disorders^40^. VNS has generally been used to treat epilepsy and was approved by the Food and Drug Administration in 2005 as a treatment for people with treatment-resistant depression or severe, recurrent unipolar and bipolar depression^29^. VNS activates the vagus afferent fibers in the neck, which carry impulses to the brainstem to target the LC and dorsal raphe nucleus. It was also reported that VNS improved the working memory performance in patients with epilepsy, as seen by the reduced errors on a subtask that relied on working memory and increased N1 amplitude measured by electroencephalography^41^. In addition, auricular VNS was reported to activate the LC and reinforce memory function^42^, as well as showing anti-inflammatory effects in depression^43^. Nevertheless, surgical operation cannot be avoided for VNS treatment, which increases the risk of infection, pain, and damage to the vagus nerve; therefore, VNS is not the first selection for treatment. In contrast, VNS by food ingredients is safer and easier to apply in daily life. The effectiveness of MHBAs on the vagus nerve in humans needs to be evaluated further in a clinical study on the balance of the sympathetic and parasympathetic nervous systems.

Besides the use of VNS in epilepsy and depression, recent studies have introduced this approach for the treatment and prevention of AD. VNS was shown to activate the noradrenaline neurons in the LC and increase the levels of noradrenaline in the cortex and hippocampus^44^. In contrast, depletion of noradrenaline by DSP-4 has been found to exacerbate memory impairment and Aβ deposition in AD transgenic mice^45,46^. The microglial Aβ phagocytosis is also lower in noradrenaline-depleted AD mice than in control AD mice^45^. It was also reported that external VNS modulated the activation of microglia in AD mice^47^. In the present study, we found a high ratio of TNF-α-producing cells and lower phagocytotic activity of Aβ in noradrenaline-depleted 5 × FAD mice, consistent with a previous report^45^. Moreover, the beneficial effects of MHBAs on memory impairment and microglial activation were attenuated by noradrenaline-depletion in 5 × FAD mice. Another study showed that suppression of microglial activation by noradrenaline was crucial for improving memory impairment in DSP-4 and LPS treated mice^48^. Our previous studies have shown that IAAs and MHBAs improve memory impairment via the activation of the vagus nerve and that this improvement is attenuated by vagotomy^13,22^. Taken together, these findings indicate that hop bitter acids containing a β-tricarbonyl moiety activate the vagus nerve and stimulate noradrenaline neurons in the LC, which contributes to the reduction of microglial activation and improvement of memory impairment.

In the present study, we also demonstrated that AHC, a model compound containing a β-tricarbonyl moiety, improved the memory impairment induced by brain inflammation. In the case of MHBA treatment, memory improvement was observed after treatment with HAIH and HAH but not after that with TCOIH-A. As shown in Fig. 1, the chemical character of TCOIH-A is different compared with that of HAH, HAIH, and IAAs. This suggests that the position of the β-tricarbonyl moiety is important for the effects of memory improvement.

Further, we showed that MHBA and AHC activate intestinal epithelia cells, increasing CCK release. CCK has been associated with neuronal activation, as intraperitoneal treatment with CCK increases cFos expression in the locus coeruleus/subcoeruleus nucleus whereas CCK knockdown leads to memory impairment, assessed by the NORT^49,50^. CCK is crucial for gut-brain communication via the vagus nerve to regulate food intake, body weight, and inflammation^51–53^. Moreover, endothelial dysfunction was found to be associated with cognitive impairment^54^. On the whole, CCK from enterocytes activates CCK receptors on the vagus nerve and the afferent vagus nerve results in the stimulation of the noradrenergic neuron in LC. LC neurons project to the hippocampus and frontal cortex and modulate the level of norepinephrine which suppresses the inflammatory responses of microglia and increases phagocytosis. In the current study, MHBA and AHC increased the CCK level from enterocytes and the memory improvement and the reduction of TNF-α production by MHBA were attenuated by the depletion of noradrenergic neuron or vagotomy. Collectively, these findings suggest that the increase in CCK levels by hop bitter acids containing a β-tricarbonyl moiety leads to vagus nerve activation and improve the cognitive impairment induce by inflammation^55^.

The present study has some limitations. We did not examine the involvement of bitter taste receptor in MHBA-mediated memory improvement in vivo; thus, further studies are needed to assess this using bitter taste receptor knock out mice. Moreover, future studies should also confirm the involvement of CCK in the effects of MHBAs in vivo. In the current study, we used DSP-4 to deplete the noradrenergic neuron, but we did not observed in detail whether DSP-4 specifically depleted the noradrenergic LC neuron or showed other effects than noradrenergic neuronal damage in mice. Therefore, further studies need to be conducted to evaluate the involvement of noradrenergic neuron on memory improvement by MHBAs. In addition, we conducted the experiments using male mice to avoid the effects of estrous cycle on behavioral analysis and inflammation; theus, to generally conclude, we need to evaluate using female mice in the further study.

## Conclusions

Our study reveals that food ingredients may prevent AD pathology and reduce inflammatory response via VNS. Activation of vagus nerve for improving cognitive function by hop bitter acids may be a practical approach in daily life. Therefore, future clinical studies are necessary to evaluate the effects of MHBAs in patients with AD.

## Supplementary information

Supplementary Information.

## Data Availability

The datasets used and/or analyzed during the current study are available from the corresponding author on reasonable request.

## Abbreviations

AD: Alzheimer’s disease
Aβ: Amyloid-β
AHC: 2-Acetyl-3-hydroxy-2-cyclopenten-1-one
APP: Amyloid precursor protein
CCK: Cholecystokinin
DG: Dentate gyrus
ECD: Electrochemical detection
ELISA: Enzyme-linked immunosorbent assay
FAD: Familial Alzheimer's Disease
HAH: 4′-Hydroxyallohumulinones
HAIH: 4′-Hydroxyalloisohumulones
HPLC: High performance liquid chromatography
IAAs: Iso-α-acids
LC: Locus coeruleus
LPS: Lipopolysaccharide
MHBAs: Matured hop bitter acids
NFTs: Neurofibrillary tangles
NORT: Novel object recognition test
TCOIH-A: Tricyclooxyisohumulones A
TNF-α: Tumor necrosis factor-α
VNS: Vagus nerve stimulation
