# Supplementary material for: Hop bitter acids containing a β-carbonyl moiety prevent inflammation-induced cognitive decline via the vagus nerve and noradrenergic system
Source: Sci Rep. 2020 Nov 18;10:20028. doi: 10.1038/s41598-020-77034-w (PMC7674441; doi:10.1038/s41598-020-77034-w)
Supplement: Supplementary file 1 — Supplementary Information. [file 41598_2020_77034_MOESM1_ESM.docx]

**Supplementary Figure S1**

**Hop bitter acids containing a β-carbonyl moiety prevent inflammation-induced cognitive decline via the vagus nerve and noradrenergic system**

Yasuhisa Ano, Rena Ohya, Takahiro Yamazaki, Chika Takahashi, Yoshimasa Taniguchi, Keiji Kondo, Akihiko Takashima, Kazuyuki Uchida, Hiroyuki Nakayama


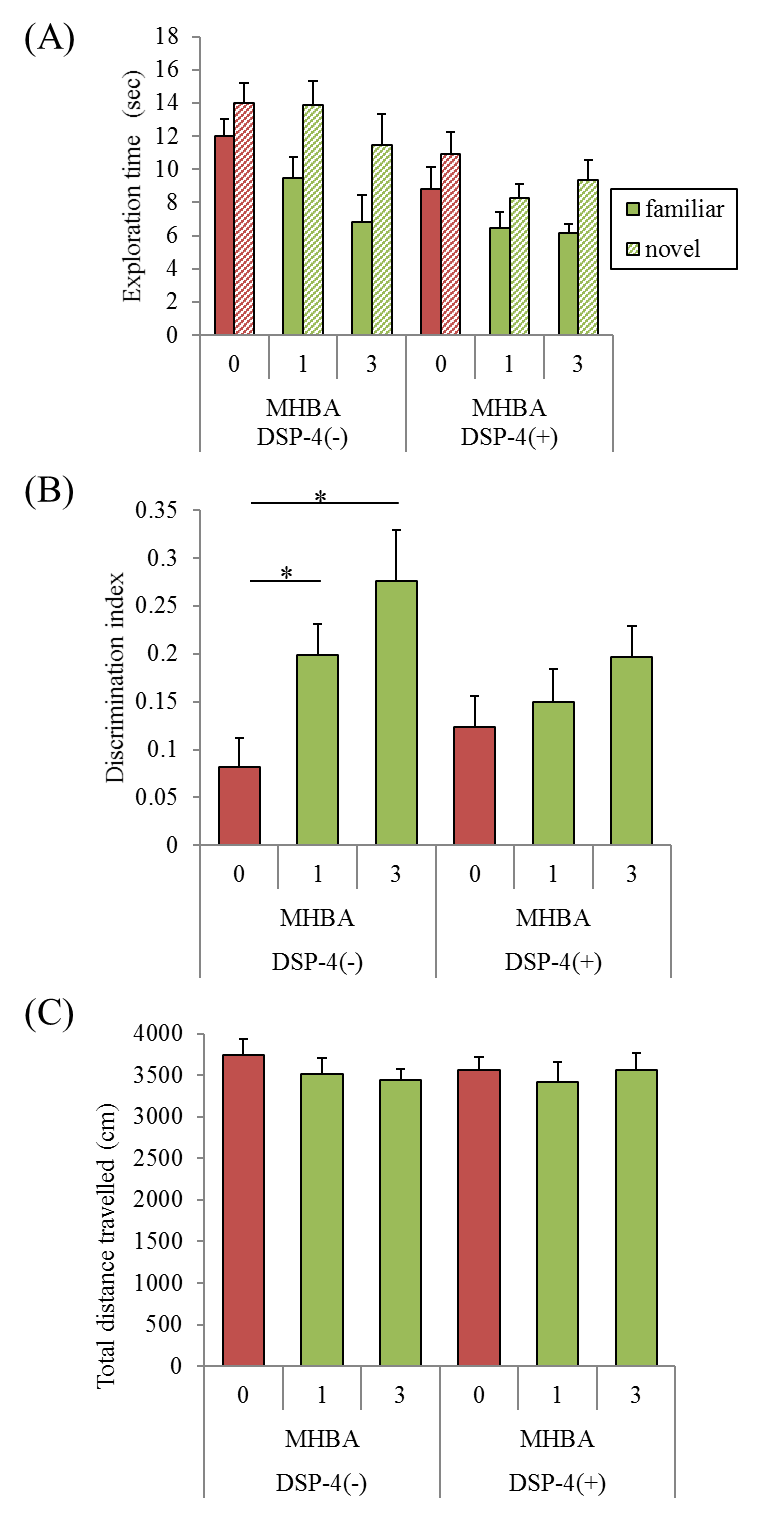


**Supplementary Figure S1 Involvement of noradrenaline in memory improvement by MHBAs**

Mice were treated intraperitoneally with saline or 50 mg/kg DSP-4, and subsequently intracerebroventricularly injected with 10 μg of lipopolysaccharide (LPS). Mice were subjected to the NORT and open field test. A, Time of exploring novel or familiar objects. B, Discrimination index in the NORT. C, Total distance travelled in the open field test (cm). Data are presented as mean ± standard error of the mean (7 mice per group). The *p* values shown were calculated by two-way analysis of variance followed by the Tukey–Kramer test. **p* < 0.05. MHBAs, matured hop bitter acids; DSP-4, N-(2-chloroethyl)-N-ethyl-2-bromobenzylamine; LPS, lipopolysaccharide; NORT, novel object recognition test
